# Essential and Non-essential Trace Elements in Milks and Plant-Based Drinks

**DOI:** 10.1007/s12011-021-03021-5

**Published:** 2021-11-18

**Authors:** Montse Marquès, Eudald Correig, Esther Capdevila, Eva Gargallo, Neus González, Martí Nadal, José L. Domingo

**Affiliations:** 1 Laboratory of Toxicology and Environmental Health, School of Medicine, Universitat Rovira i Virgili, IISPV, Sant Llorenç 21, 43201 Reus, Catalonia Spain; 2 Department of Biostatistics, School of Medicine, Universitat Rovira i Virgili, Sant Llorenç 21, 43201 Reus, Catalonia Spain

**Keywords:** Cow milk, Goat milk, Plant-based drinks, Essential elements, Toxic elements

## Abstract

Although milk and plant-based drinks are widely consumed foodstuffs with high nutritional value, their consumption may also mean intake of non-essential/toxic elements becoming a risk for human health. This study was aimed at determining the concentrations of essential (Ca, Co, K, Mg, Mn, Na, Ni and P) and non-essential/toxic (Hg, Pb, U and V) elements in milks (cow and goat), plant-based drinks (soy, almond, rice and oat) and infant formulas from organic and conventional production systems. Lactose-free, fresh and ultra-high-temperature (UHT) milks were also included. Chemical analyses were performed by means of inductively coupled plasma-mass spectrometry (ICP-MS). The content of the elements hereby assessed did not depend on the production system and the presence of lactose. However, significant differences were found in the concentrations of multiple elements when comparing sterilization methods, source (animal vs. plant-based) and animal species. Non-essential elements were not detected in milks and plant-based drinks, excepting Pb, which was detected in three samples. While the consumption of goat milk is recommended, considering the global intake of essential elements and the absence of non-essential elements, further studies should be conducted to confirm the absence of non-target toxic elements at very low trace levels. On the other hand, the best plant-based drinks are those made up with almonds (intake of Ca) and soy (K and Mg). The current results should be useful to help the population to balance the benefits and risks from milks and plant-based drinks consumption, as well as to adapt their dietary habits.

**Supplementary Information:**

The online version contains supplementary material available at 10.1007/s12011-021-03021-5.

## Introduction

Milk is a nutritious liquid food excreted by the mammary glands of mammals, which is mainly composed of water, carbohydrates (lactose), proteins (casein), fatty acids (triacylglycerides, diacylglycerides, saturated and polyunsaturated fatty acids and phospholipids), vitamins (mainly retinol, thiamine, riboflavin and niacin) and a number of trace elements [1–3]. Milk contains many biologically essential elements, but it may also contain non-essential/toxic trace elements [2, 4]. Elements such as calcium (Ca), chrome (Cr), cobalt (Co), copper (Cu), iron (Fe), potassium (K), magnesium (Mg), manganese (Mn), molybdenum (Mo), sodium (Na), nickel (Ni), phosphorus (P), selenium (Se) and zinc (Zn) are essential, playing a basic role in the maintenance of biochemical and physiological functions in living organisms. Their role depends on each specific element. For example, Ca is involved in vascular, endocrine and neuromuscular function [5], while Cu and Fe are involved in physiological balance (i.e. homeostasis) [6–8]. K is involved in vascular function (i.e. blood pressure regulation) [9], Mg in endocrine function (i.e. blood glucose control) and biochemical reactions [10] and Mn in immune function and in physiological balance (i.e. homeostasis, coagulation) [7, 11]. In turn, Na is involved in vascular and neuromuscular function (i.e. transmission of nerve impulses) [9], P in multiple physiological functions [12], while Zn is involved in many biochemical and enzymatic reactions [7]. However, if certain concentration thresholds of these elements are exceeded, they may generate homeostatic disturbances, being able of bioaccumulating and biomagnifying in the body, leading to the formation of free radicals, oxidative stress disorders, and consequently, becoming harmful for human health [13, 14].

In turn, non-essential elements do not have any known function in the human body and they might be toxic even at low concentrations [15, 16]. Among non-essential trace elements, there are heavy metals and metalloids such as aluminium (Al), arsenic (As), cadmium (Cd), mercury (Hg), lead (Pb), antimony (Sb), tin (Sn), uranium (U) and vanadium (V). Their toxicities are related to their capability to damage vital organs such as the brain, kidney or liver, among others [17]. Long-term exposure to non-essential elements may lead to physical (i.e. chronic pain, blood pressure alteration, blood composition change, etc.) and psychological (i.e. anxiety, passivity, etc.) disorders, neurodegenerative diseases and cancer [17, 18]. Regarding the latter, inorganic As and Cd are classified as “carcinogenic to humans (Group 1)” by the IARC [19].

Heavy metals and metalloids are natural components of the Earth’s crust [17]. However, the industrialization, the high-energy demand and the exploitation of natural resources have increased their environmental occurrence [20]. After their emissions from the sources, they can be transported and deposited on the soil [20, 21]. Hence, livestock is exposed to these elements, which enter into the trophic chain through consumption of their meat or dairy products, such as milk [22], becoming potential health risks [23, 24].

The presence of trace elements in milk — and its composition — can vary due to factors such as climate, season of production and origin country and breed of the producing animal, among others [25–27]. Most studies regarding this issue have assessed the influence of the aforementioned parameters on the content of a few essential and non-essential elements. However, these studies did not analyse the wide range of milks available for consumption in food stores and supermarkets. Furthermore, and due to the changes in lifestyles, an aversion to animal cruelty and an increasing environmental awareness, in recent years, some consumers have decided to substitute dairy milks by plant-based drinks [28]. The occurrence of essential and non-essential elements in plant-based drinks is less explored than their levels in milks. To the best of our knowledge, there is a gap in the comparison between the concentrations of these elements in animal and plant-based drinks [29–31]. Therefore, the present study was aimed at assessing the concentrations of essential elements (Ca, Co, K, Mg, Mn, Na, Ni and P) and non-essential elements (Hg, Pb, U and V) in milks (cow and goat), plant-based drinks (soy, almond, rice and oat) and infant formulas from organic and conventional production systems. Lactose-free, fresh and ultra-high-temperature (UHT) milks were also included when available in the Spanish markets. In the current study, we determined whether the content of essential and non-essential elements depends on the production system (conventional or organic), origin (animal or plant-based), animal source (cow or goat), sterilization method (fresh or UHT) and the presence (or absence) of lactose. We also identified the best type of milk in terms of benefits (intake of essential elements) and risks (intake of non-essential elements).

## Materials and Methods

### Sampling

In January 2021, milks and plant-based drinks were acquired in various supermarkets located in Reus (Catalonia, Spain). The most consumed types of milks and plant-based drinks were selected according to the results of ENALIA surveys (National Survey of Nutrition in the Child and Adolescent Population of Spain) [32], including conventional and organic farming systems, lactose-free, fresh and ultra-high-temperature (UHT) milks. These are as follows: cow milk (whole, organic whole, semi-skimmed, organic semi-skimmed, skimmed, organic skimmed, lactose-free, organic lactose-free, whole fresh, organic whole fresh, skimmed fresh, organic skimmed fresh, skimmed fresh); goat (semi-skimmed, organic whole, whole fresh, organic whole fresh); soy milk and organic soy milk; almond milk and organic almond milk; rice milk and organic rice milk; oat milk and organic oat milk; and infant formula and organic infant formula. Triplicates of each dairy and plant-based drink were obtained when up to three different brands were available.

### Sample Treatment

Composites of each milk and plant-based drink were done with 5 mL of each individual sample. For infant formula powder, 15 g was mixed with 30 mL of purified water, and subsequently, the composite was made up with 5 mL of each sample. Composites were immediately placed into vials and frozen at − 20 °C until further analysis.

Up to 5 µL of each sample was mixed with 5 mL of HNO_3_ (10%, Suprapur, E. Merck, Darmstadt, Germany) in hermetic Teflon vessels during 8 h at room temperature (pre-digestion) and 8 h at 80 °C. Once the digestion was completed, samples were cooled at room temperature. Extracts were then filtered and made up to 25 mL with purified water [13, 33]. Samples were analysed in duplicate to achieve an acceptable accuracy in the results.

### Analytical Procedure and Quality Control

The concentrations of Ca, Co, Hg, K, Mg, Mn, Na, Ni, P, Pb, U and V were determined by inductively coupled plasma-mass spectrometry (ICP-MS). The analytical methods were previously described [33–35]. Spinach leaves (SRM 1570a) and whole milk powder (SRM 1549a) certified by the National Institute of Standards and Technology were used as standard reference materials. Spinach leaves were used to determine the recoveries of Mn, Hg, Pb, Ni, V, Co and U, while whole milk powder was used for K, Ca, Mg, Na and P. Recoveries were between the range 75 and 110%. Only Ni showed a slightly low recovery (55%). The limits of detection (LD) were set at 250 μg/g for K, Na and P, 50 μg/g for Ca, 25 μg/g for Mg, 0.05 μg/g for Hg, Mn, Ni, Pb and V, and at 0.025 μg/g for Co and U.

### Statistics

Data are presented as median values and 25th and 75th percentiles for continuous variables with a non-normal distribution, or as the mean and standard deviation (SDs) for variables with a normal distribution. Categorical variables are reported as percentages. Continuous variables were tested for normality using the Shapiro–Wilk test. Differences between groups were analysed using the non-parametric Mann–Whitney U test, or the Welch’s parametric *t* test for continuous variables, and the chi-square test or Fisher’s exact test for categorical variables. In the significant associations, we measured the strength of the observed effect (i.e. the effect size) with Cohen’s d method.

An unsupervised clustering procedure was used to understand the similarities and differences among the different types and sources of the studied milks. We used a k-means clustering method and chose the number of clusters (3) using the elbow method. A principal component analysis (PCA) was performed to visualize the relationship between such clusters and each of the studied elements with a significant detection rate.

Confidence intervals were given with a 95% confidence, being type I error set at 5%. Metals not showing enough observations above the respective LD were not included in the analysis. All statistical analyses were performed using the R software package version 4.0 [36] along with SPSS 27.0 [37].

## Results and Discussion

### Concentrations of Essential and Non-essential Elements in Milks and Plant-Based Drinks

Table 1 shows the concentrations of essential and non-essential elements in milks and plant-based drinks here analysed. It can be seen that all drinks contained Ca, K, Mg, Na and P. In turn, Mn and Ni were also detected, but in a lower frequency (19 and 6 out of 32 samples, respectively). Finally, Co was not detected in any of the milk and plant-based drinks samples. The highest levels of essential elements were found in conventional soy-based drink (Mg and Mn), non-organic skimmed fresh cow milk (Ni), organic whole fresh goat milk (Ca), organic whole goat’s milk (K and Na) and non-organic semi-skimmed goat milk (P).

**Table 1:** Concentrations (µg/kg) of a number of essential and non-essential elements in milks and plant-based drinks

	Hg	Pb	U	V	Ca	Co	K	Mg	Mn	Na	Ni	P
Whole cow’s milk	< LD	0.027	< LD	< LD	1084	< LD	1485	108	0.035	388	< LD	905
Organic whole cow’s milk	< LD	< LD	< LD	< LD	1058	< LD	1493	104	0.078	375	< LD	830
Semi-skimmed cow’s milk	< LD	< LD	< LD	< LD	1110	< LD	1537	110	< LD	408	< LD	931
Organic semi-skimmed cow’s milk	< LD	< LD	< LD	< LD	1086	< LD	1551	109	< LD	393	< LD	855
Skimmed cow’s milk	< LD	0.033	< LD	< LD	1083	< LD	1509	108	0.064	416	< LD	927
Organic skimmed cow’s milk	< LD	< LD	< LD	< LD	1139	< LD	1508	106	< LD	391	< LD	875
Lactose-free cow’s milk	< LD	< LD	< LD	< LD	1085	< LD	1518	108	< LD	395	< LD	919
Organic lactose-free cow’s milk	< LD	< LD	< LD	< LD	1133	< LD	1505	105	< LD	349	< LD	881
Organic whole goat milk	< LD	< LD	< LD	< LD	1010	< LD	1754	139	< LD	657	< LD	1010
Semi-skimmed goat milk	< LD	< LD	< LD	< LD	1245	< LD	1513	142	< LD	591	< LD	1105
Whole fresh cow’s milk	< LD	< LD	< LD	< LD	1093	< LD	1462	109	< LD	345	< LD	858
Organic whole fresh cow’s milk	< LD	< LD	< LD	< LD	1108	< LD	1448	109	< LD	334	< LD	917
Skimmed fresh cow’s milk	< LD	< LD	< LD	< LD	1066	< LD	1459	106	< LD	327	< LD	871
Organic skimmed fresh cow’s milk	< LD	< LD	< LD	< LD	1093	< LD	1463	103	< LD	318	< LD	878
Skimmed fresh cow’s milk	< LD	< LD	< LD	< LD	1116	< LD	1512	112	< LD	344	0.435	907
Whole fresh goat milk	< LD	< LD	< LD	< LD	1159	< LD	1265	146	< LD	326	< LD	985
Organic whole fresh cow’s milk	< LD	< LD	< LD	< LD	1343	< LD	1373	147	< LD	337	< LD	1033
Almond drink	< LD	< LD	< LD	< LD	1064	< LD	140	62	0.420	463	< LD	367
Organic almond drink	< LD	< LD	< LD	< LD	327	< LD	239	88	0.456	295	< LD	141
Oat drink	< LD	0.220	< LD	< LD	236	< LD	341	39	0.665	135	< LD	240
Organic oat drink	< LD	< LD	< LD	< LD	324	< LD	292	42	0.671	312	0.089	148
Soy drink	< LD	< LD	< LD	< LD	682	< LD	1473	184	2.052	242	0.330	735
Organic soy drink	< LD	< LD	< LD	< LD	139	< LD	1146	134	1.444	198	0.283	278
Rice drink	< LD	< LD	< LD	< LD	447	< LD	143	21	0.138	244	< LD	299
Organic rice drink	< LD	< LD	< LD	< LD	270	< LD	121	40	0.354	445	< LD	71
Follow-on formula milk	< LD	< LD	< LD	< LD	366	< LD	782	46	0.128	148	< LD	271
Organic follow-on formula milk	< LD	< LD	< LD	< LD	357	< LD	643	53	0.102	204	< LD	300
Follow-on formula milk 2	< LD	< LD	< LD	< LD	545	< LD	821	61	0.169	232	0.049	363
Organic follow-on formula milk 2	< LD	< LD	< LD	< LD	548	< LD	676	58	0.169	171	0.094	373
Follow-on formula milk 3	< LD	< LD	< LD	< LD	1036	< LD	822	51	0.126	172	< LD	513
Organic follow-on formula milk 3	< LD	< LD	< LD	< LD	631	< LD	949	80	0.080	204	< LD	445
Organic follow-on formula milk 4	< LD	< LD	< LD	< LD	638	< LD	788	97	0.162	184	< LD	343
*Limit of detection (LD)*	0.05	0.05	0.025	0.05	50	0.025	250	25	0.05	250	0.05	250

Regarding non-essential/toxic elements, Pb was detected in non-organic whole cow milk, skimmed cow milk and non-organic oat drink. Although the concentration of the Pb was below the limit of quantification (LQ) in non-organic whole and skimmed cow milk, its level exceeded the maximum limit set by the Codex Alimentarius (CODEX STAN 193–1995) at 0.02 µg/kg in these samples. In contrast, Hg, U and V were not detected in any of milks and plant-based drinks hereby analysed.

### Comparison Between Groups

Those elements detected with a high frequency (> 50%) (Ca, K, Mg, Mn, Na, P) in milks and plant-based drinks were compared for production systems (conventional vs. organic), sterilization methods (fresh vs. UHT), source (animal vs. plant-based), animal species (cow vs. goat), presence of lactose (lactose vs. lactose-free) and production system of infant formula (conventional vs. organic).

#### Production System

No significant differences (*p* > 0.05) were found between the occurrence of Ca, K, Mg, Mn, Na and P (Supplementary Information, Fig. S1) in conventional and organic milks and plant-based drinks. These results are in agreement with those of previous studies [38, 39], as well as with the fact that the organic certification does not include the occurrence of essential and non-essential elements in food.

#### Sterilization Method

**Fig. 1:**
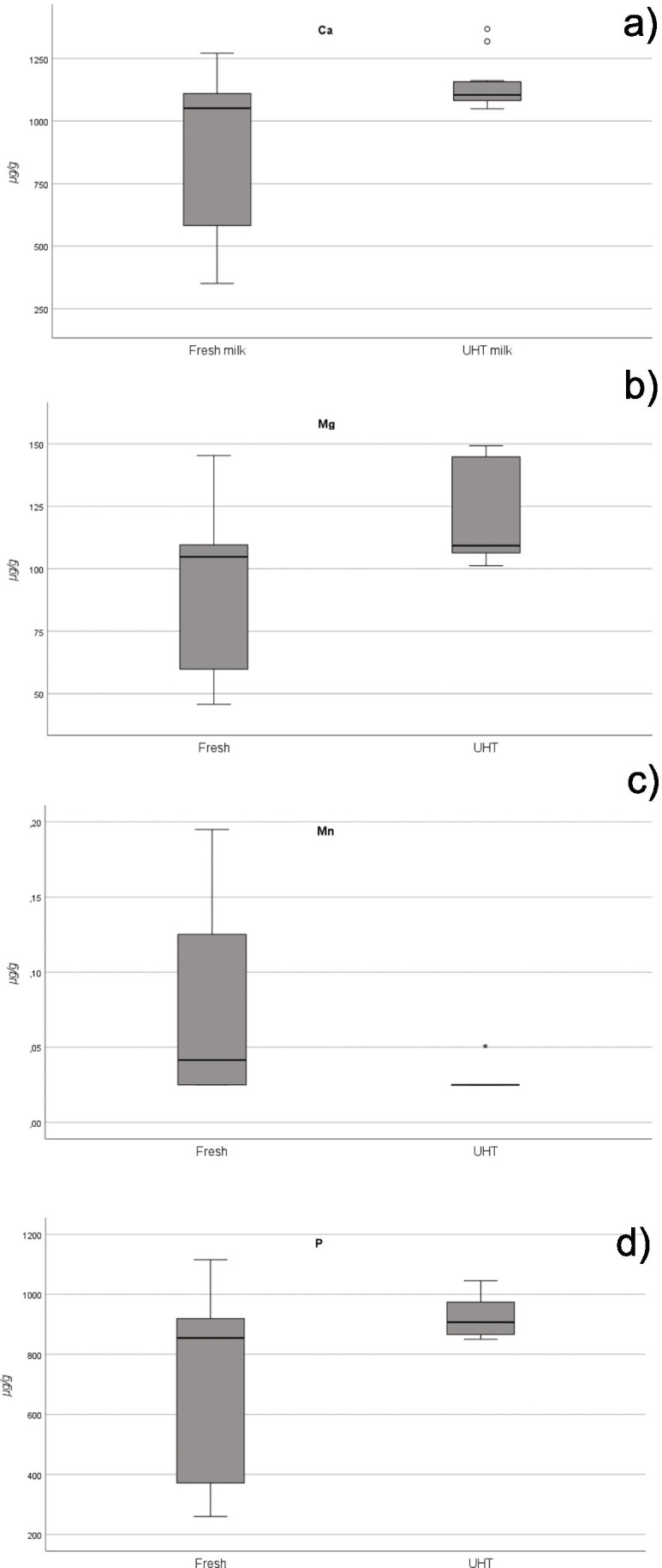
Box plots of Ca (**a**), Mg (**b**), Mn (c) and P (**d**) concentrations in milk sample according to sterilization method (fresh or UHT)

Only milks were included. The occurrence of Mn was significantly higher (*p* < 0.05) in UHT than in fresh milks (effect size measured by Cohen’s d, 95% CI of 1.00 (0.34, 1.65) (Fig. 1c). In contrast, the content of Ca, Mg and P (Fig. 1a, b, d) was higher (*p* < 0.05) in fresh than in UHT milks, and the effect size is measured by Cohen’s d, 95% CI of − 0.97 (− 1.62, − 0.32), − 0.951 (− 1.60, − 0.30), − 0.92 (− 1.56, − 0.26). Differences between the concentrations of the remaining essential elements (K and Na) (Supplementary Material, Fig. S2) were not statistically significant (*p* > 0.05). Our findings are not in accordance with those of Guney et al. [40], who reported that heat treatment did not change the content of Ca, K, Mg, Na and P in milk, and those of Singh et al. [41] who demonstrated that the content of Ca and P increased with the application of a heat treatment.

#### Origin (Animal- or Plant-Based)

**Fig. 2:**
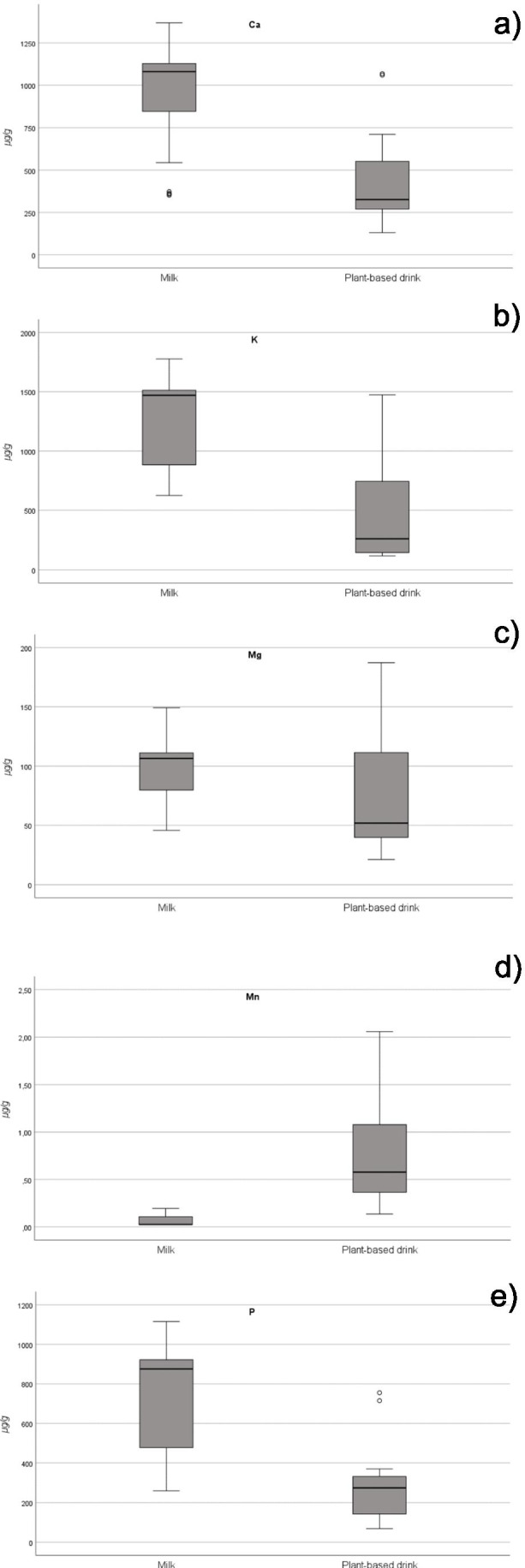
Box plots of Ca (**a**), K (**b**), Mg (**c**), Mn (**d**) and P (**e**) concentrations in milks and plant-based drinks

The levels of Ca, K, Mg and P (Fig. 2a, b, c, e) were significantly higher (*p* < 0.05) in milks than in plant-based drinks (effect size measured by Cohen’s d, 95% CI of 1.88, (1.22; 2.53), 2.07 (1.39; 2.74), 0.66 (0.08; 1.23) and 1.92 (1.25; 2.57), respectively). In turn, the content of Na was higher in milks than in plant plant-based drinks (Supplementary Material, Fig. S3). However, the differences were not significant (*p* > 0.05). By contrast, the occurrence of Mn (Fig. 2d) was significantly higher (*p* < 0.05) in plant-based drinks than in animal milks (effect size measured by Cohen’s d, 95% CI of − 2.30 (− 2.99; − 1.60)).

Dairy products have been already identified as significant sources of Ca, K, Mg, Na and P [5, 29]. Moreover, Astolfi et al. [29] reported that Ca, K and P were the elements occurring at the highest concentrations in milks, while Mn was the most abundant element in plant-based drinks, especially in soy beverages, being in agreement with the current findings. On the contrary, Dávila de Campagnaro [30] reported that almond drink was rich in Mg and P, having higher Ca concentrations than cow’s milk. This is not in agreement with the results of the current study and those of Astolfi et al. [29], but we found that the soy drink has the highest content of Mg. Finally, Dávila de Campagnaro [30] found that rice and oat drinks had a low content of Ca, while we observed that among all the plant-based drinks, the almond drink was the richest in Ca.

#### Animal Species

**Fig. 3:**
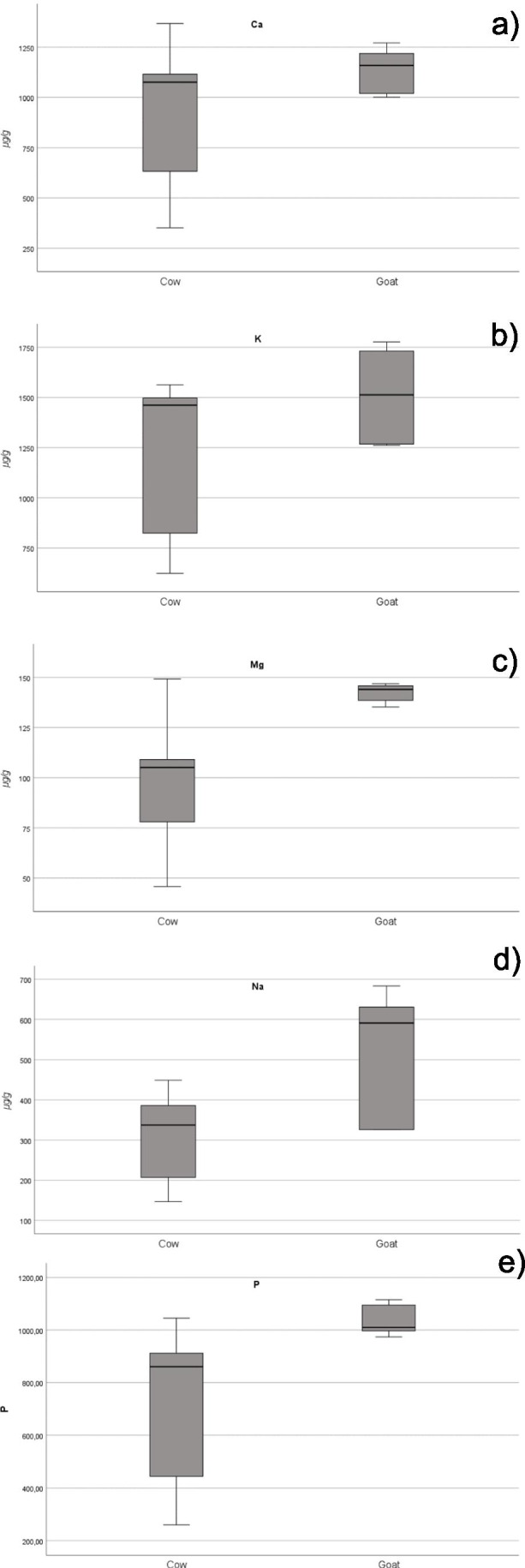
Box plots of Ca (**a**), K (**b**), Mg (**c**), Na (**d**) and P (**e**) concentrations in cow and goat milk

The concentrations of Ca, K, Mg, Na and P (Fig. 3a, b, c, e, f) were significantly higher (*p* < 0.05) in goat than in cow milk (effect size measured by Cohen’s d 95% CI of − 0.73 (− 1.60; 0.14), − 0.78 (− 1.64; − 0.95) and − 1.93 (− 2.86; − 0.98), − 2.18 (− 3.13; − 1.21), − 1.27 (− 2.15; − 0.37), respectively). In contrast, no significant differences were detected between the concentrations of Mn in goat and cow milks (Supplementary Material, Fig. S4).

Our findings are in agreement with those of Lopez et al. [42], who reported a significantly (*p* < 0.05) higher content of Mg and P in goat than in cow milk. Anyhow, these authors also noticed that cow milk had already been reported as a good source of Mg and P, taking into account the estimated recommended daily allowances.

#### Presence of Lactose

Vegetable drinks were excluded from this analysis because of their plant-based source. Present results did not show significant differences (*p* > 0.05) in the concentrations of Ca, K, Mg, Mn, Na and P between regular milk and lactose-free milk (Supplementary Material, Fig. S5). These results are in agreement with those reported by Dekker et al. [43], who did not find that the content of essential and non-essential elements depended on the presence of lactose. However, although lactose might increase the bioavailability of Ca, further studies are required to confirm this mechanism [43, 44].

#### Production System of Infant Formula

Although a previous study reported higher levels of Ca in organic than in conventional infant formulas [45], in the current study, significant differences were not found between the concentrations of essential and non-essential elements (Supplementary Material, Fig. S6) in terms of the production system (conventional or organic) in infant formulas.

#### Multivariate Analysis of Results

**Fig. 4:**
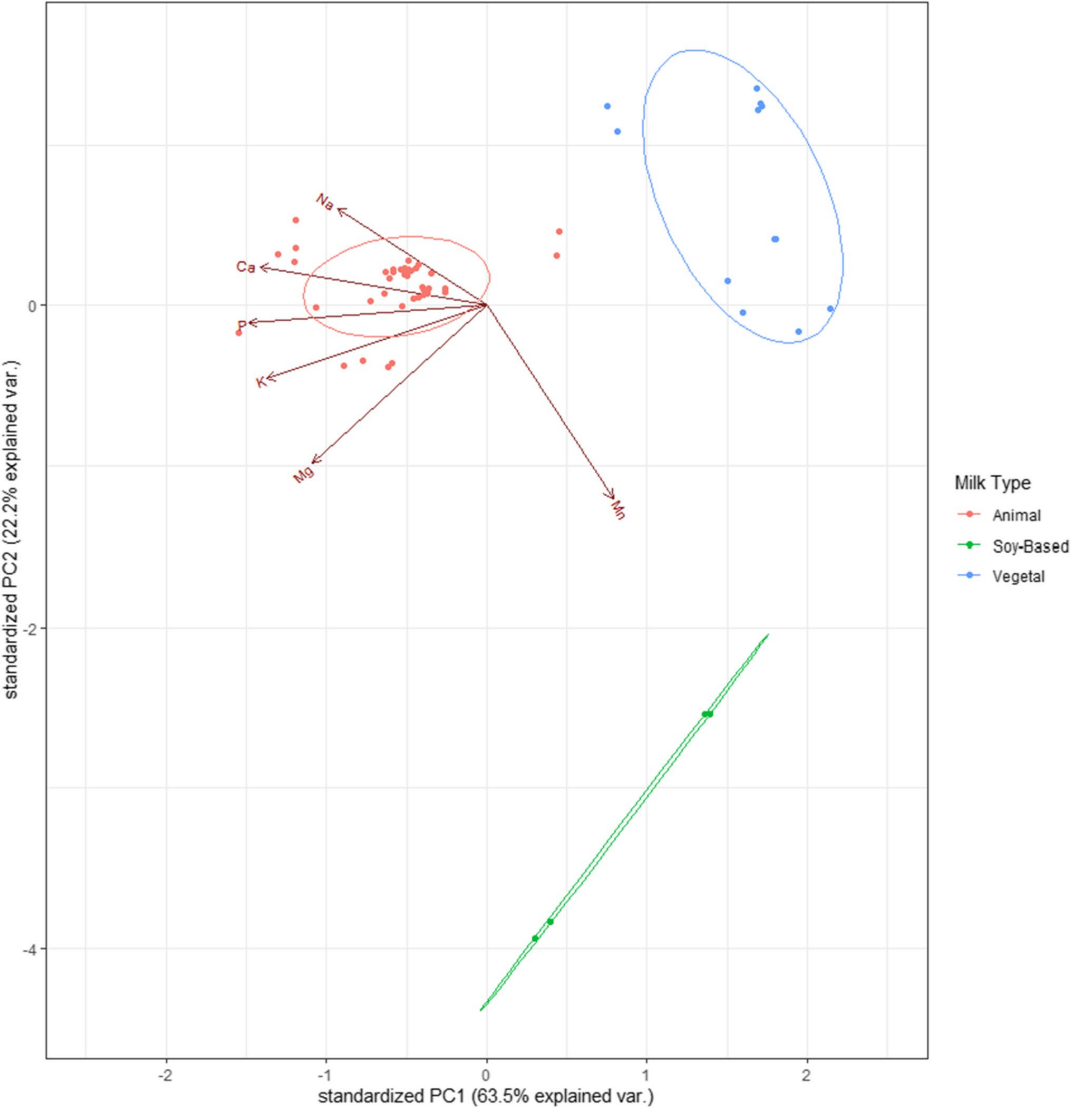
Principal component analysis of essential elements (Ca, K, Mg, Mn, Na, P)

The PCA allowed to verify the significant differences observed through the k-means clustering. Distinctions were visually appreciated in three clusters depending on the amounts of various essential elements (Ca, K, Mg, Mn, Na, P). Those elements below the respective LD (Hg, U and V), or with a detection rate below 50% (Ni and Pb), were discarded. Due to the large difference in the concentrations of elements between the soy-based drinks and the other plant-based drinks, these two were not included in the same group, being separated in 2 clusters. The three clusters are the following: (1) milks, (2) soy-based drinks and (3) the rest of plant-based drinks (Fig. 4). PCA also demonstrated that milks have a higher content of essential elements (mainly Na, Ca, P, K and Mg) than plant-based drinks, which is in accordance with the results of other studies [27, 46]. It should be highlighted the relevant intake of Mn that soy-based drinks represent, when compared to milks and other plant-based drinks. Soy-based drinks would be the best choice among all the non-animal drinks here assessed.

## Conclusions

Milks and plant-based drinks are essential components of the diet of a large part of the population. Most studies have been focused on the determination of essential and non-essential elements in raw milk directly collected from the farm [47–53]. To the best of our knowledge, there are very few studies comparing the content of essential and non-essential elements in different types of milks, plant-based drinks and infant formula products found in the market. Likewise, the impact of organic and conventional production systems, sterilization methods and the presence of lactose on their concentrations have been scarcely assessed. The results of the current study provide evidence on which type of milk or plant-based drink contains the highest concentration of each essential element. In relation to this, cow milk is rich in Ca, goat milk has the highest concentrations of K, Na and P, while soy drink those of Mg and Mn. The detection rate of Ni was very low, although cow milk and soy drink showed the highest levels. The presence of non-essential/toxic elements (Hg, Pb, U and V) was hardly appreciated in dairy milks and plant-based drinks, which means that the potential health risks derived from their consumption would be certainly low. An exception would be the detection of Pb in two samples of cow milk and one of oat-based drink, which exceeded the maximum limit established by the Codex Alimentarius (CODEX STAN 193–1995); therefore, it should be more deeply assessed.

In terms of the contents of trace elements (essential and anon-essential/toxic), milk would be the best choice among all the types of milks and plant-based drinks here examined. Goat’s milk would be the best option when balancing the human health benefits and risks. Soy drinks are recommended to avoid consuming animal products, or for those individuals who have allergies or intolerances to milks, or also in order to increase the intake of some specific elements such as Mg and Mn. Among the rest of plant-based drinks, consuming almond drinks means an intake of Ca similar to that of the milks here analysed. Anyhow, subjects who would rather consume oat or rice drinks have the chance to enhance the intake of these essential elements through other foodstuffs.

The results of the current study allow to recommend both conventional and organic production systems, as well as regular or lactose-free milks. However, further studies are required to confirm the potential role of the sterilization methods on the content of essential elements, while the absence of Pb and other non-target toxic elements should be also assessed. Finally, recommendations derived from this research can help the population to balance the benefits and risks from milk and plant-based drink consumption and to make decisions in order to improve their dietary habits.

## Supplementary Information

Below is the link to the electronic supplementary material.

Supplementary file1 (DOCX 206 KB)

## Data Availability

Results are available to any researcher upon direct request to the corresponding author.
