# Supplementary material for: Essential and Non-essential Trace Elements in Milks and Plant-Based Drinks
Source: Biol Trace Elem Res. 2021 Nov 18;200(10):4524–33. doi: 10.1007/s12011-021-03021-5 (PMC9439980; doi:10.1007/s12011-021-03021-5)
Supplement: Supplementary file 1 — Supplementary file1 (DOCX 206 KB) [file 12011_2021_3021_MOESM1_ESM.docx]

**Supplementary Information**
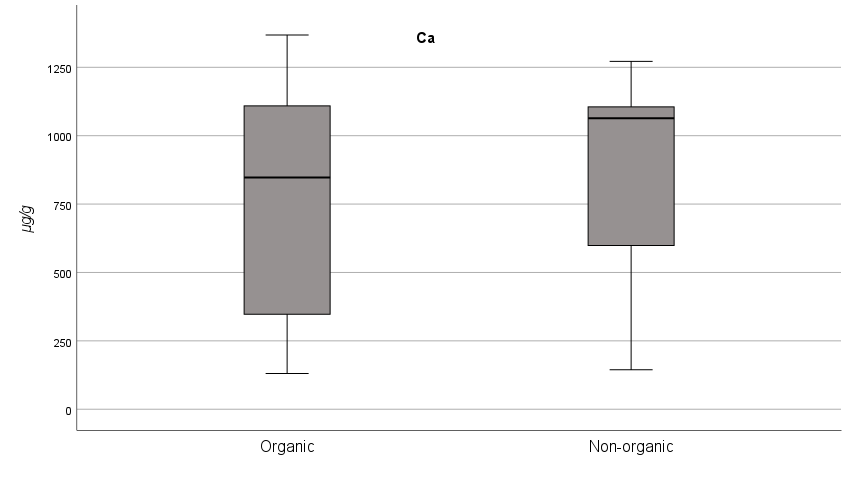


a)


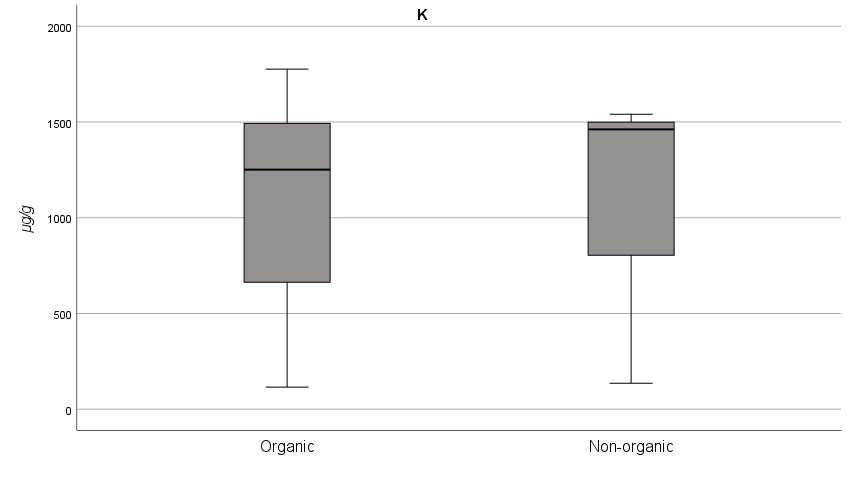


b)


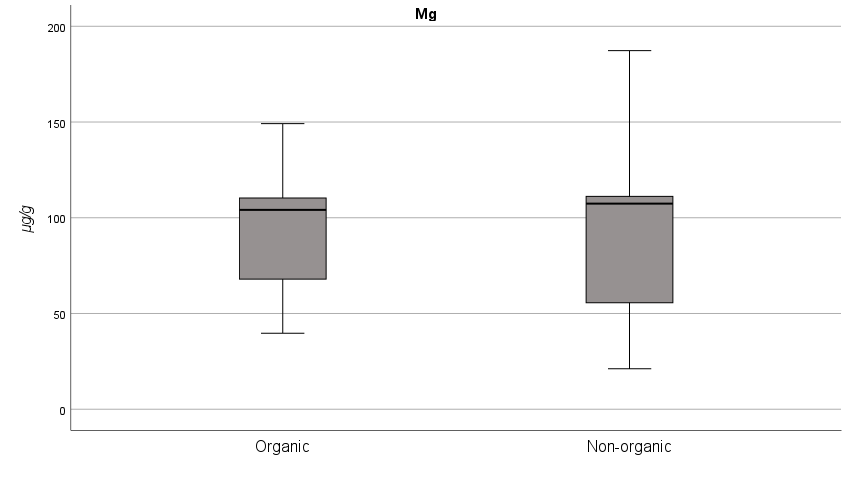


c)


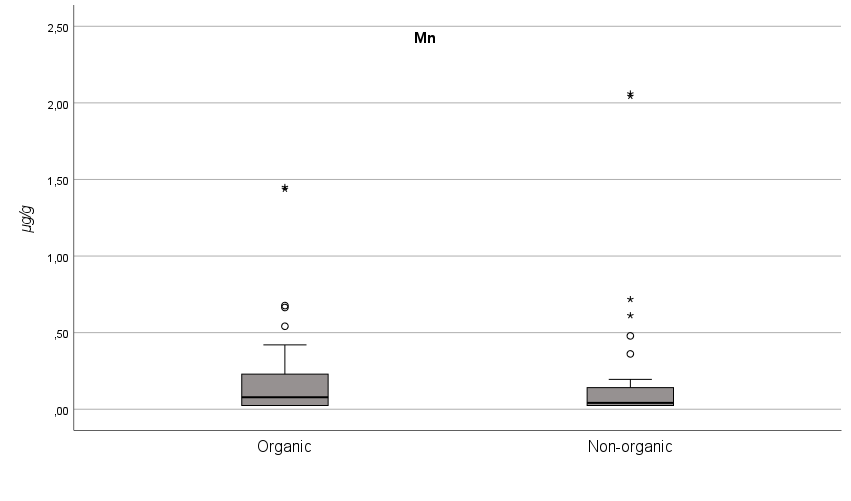


e)

d)


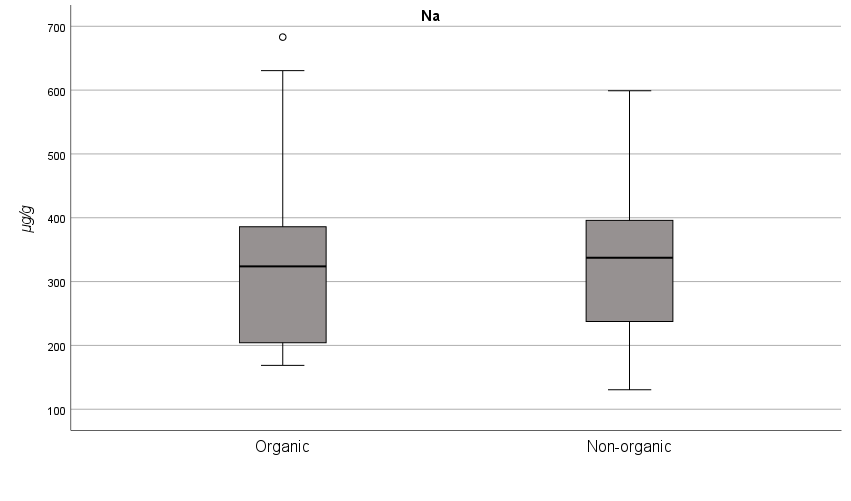


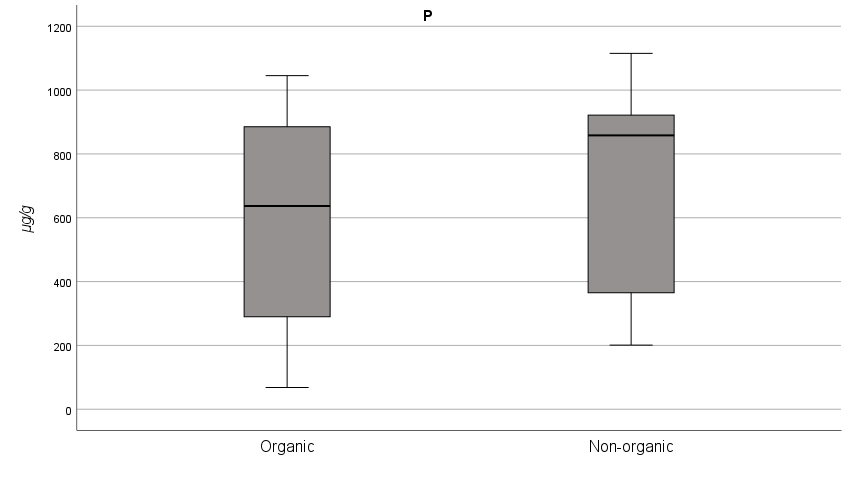


f)

**Figure S1.** Box plots Ca (a), K (b), Mg (c), Mn (d), Na (e), P (f) concentrations in milk and plant-based drinks according to the production system (organic or non-organic).


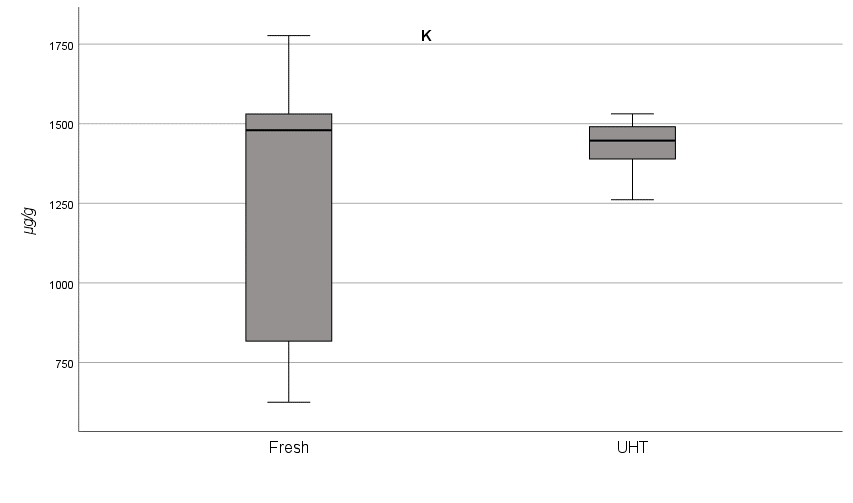


a)


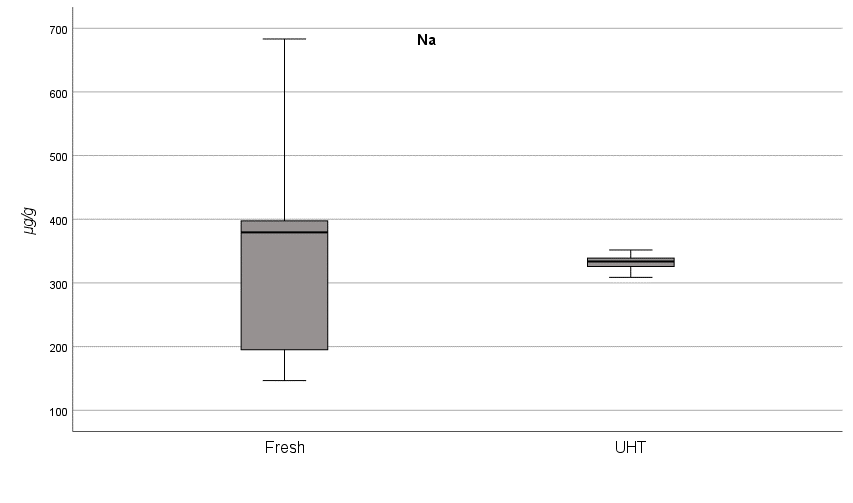


b)

**Figure S2.** Box plots K (a) and Na (b) concentrations in milk sample according to sterilization method (fresh or UHT).


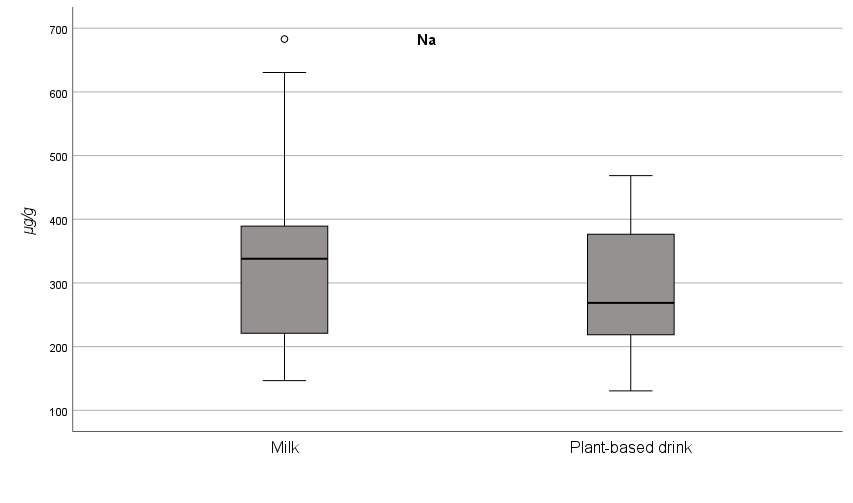


**Figure S3.** Box plot of Na concentrations in milks and plant-based drinks.


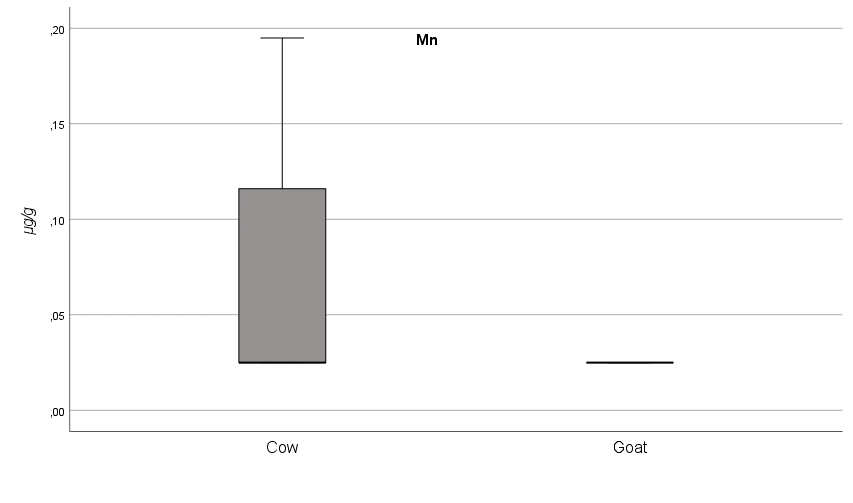


**Figure S4.** Box plot of Mn concentrations in cow and goat milks.


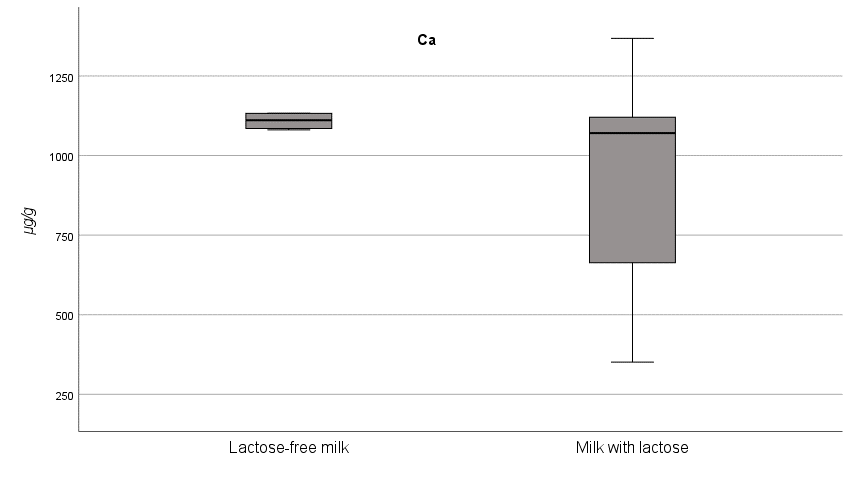


b)

a)


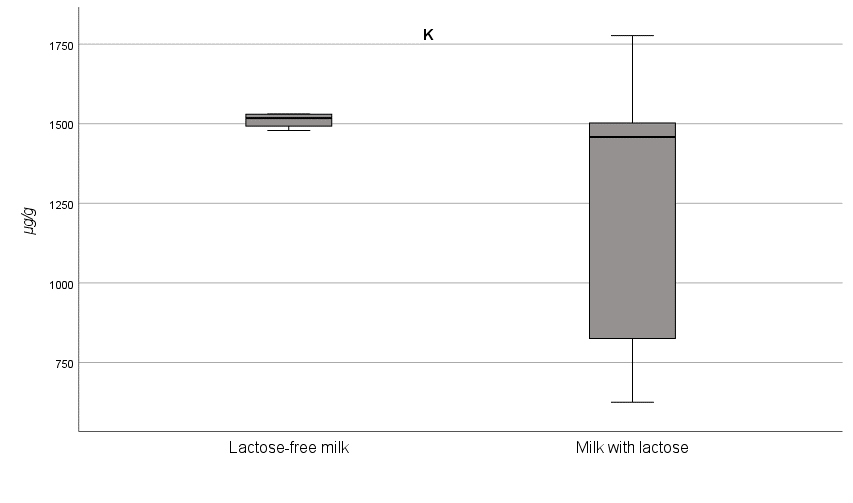


c)


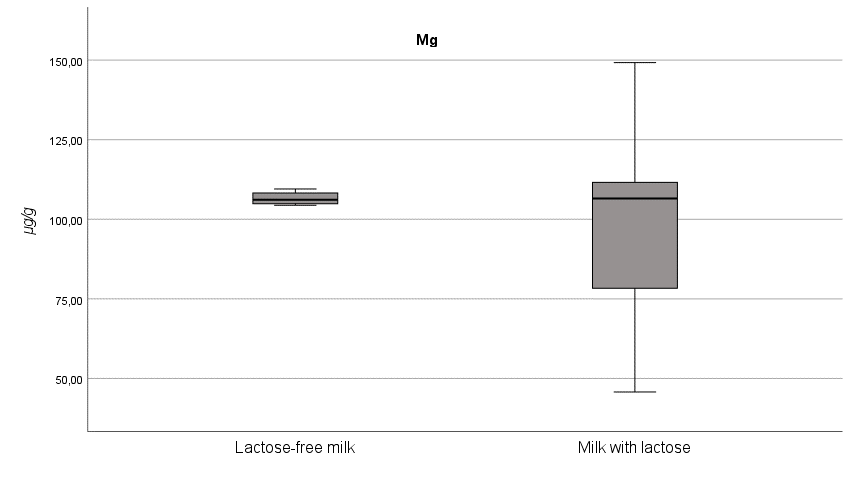


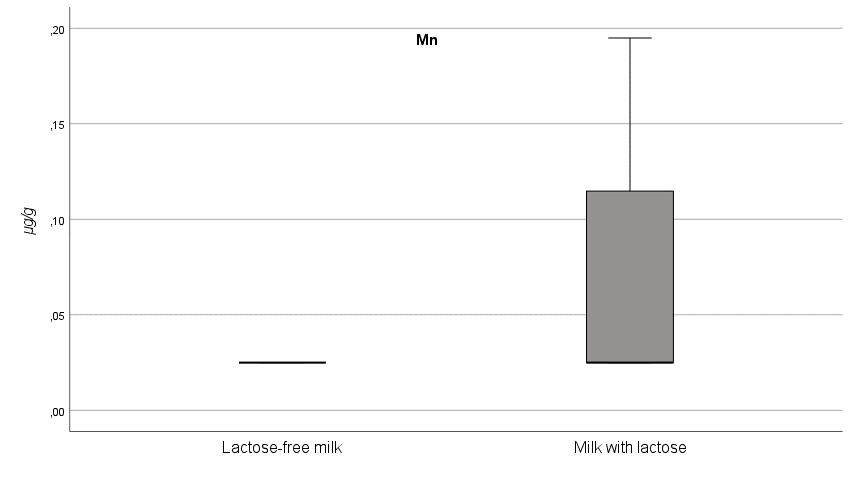


e)

d)


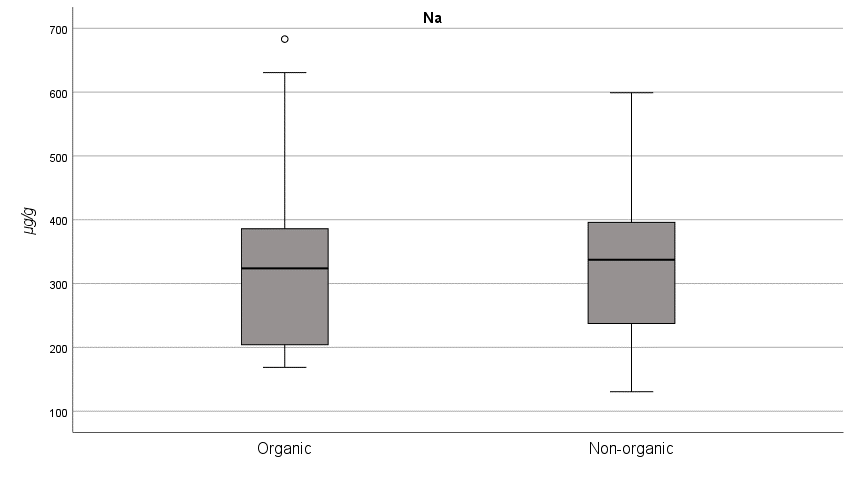


Lactose-free milk

Milk with lactose

f)


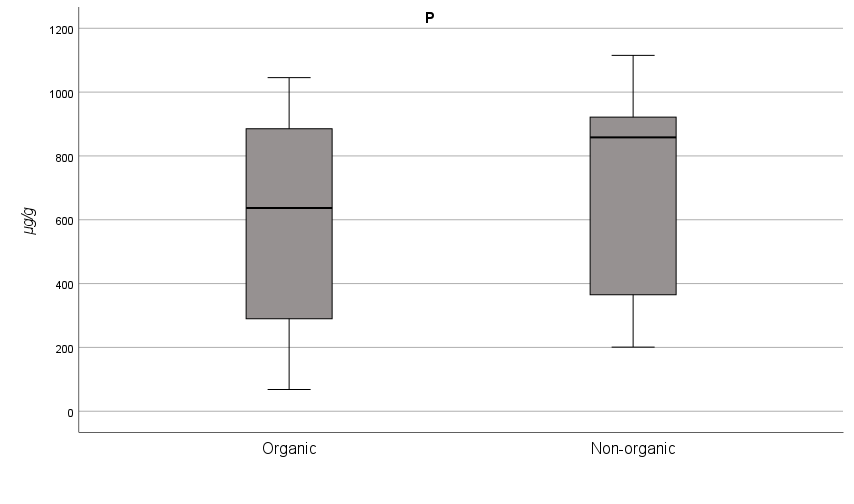


Milk with lactose

Lactose-free milk

Lactose-free

**Figure S5.** Box plots Ca (a), K (b), Mg (c), Mn (d), Na (e), P (f) concentrations in lactose and lactose-free milks.


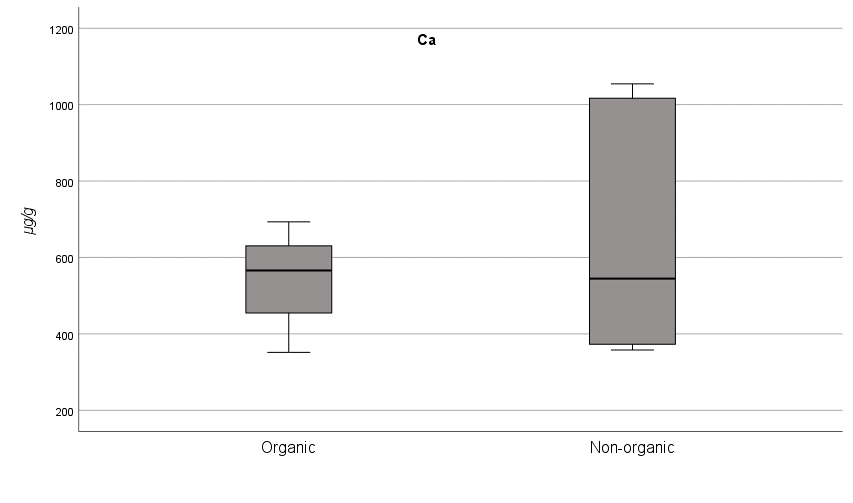


b)

a)


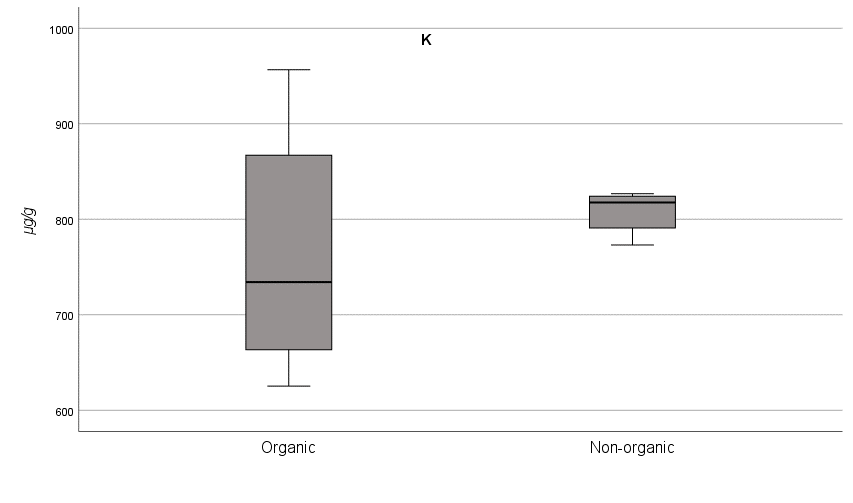


c)


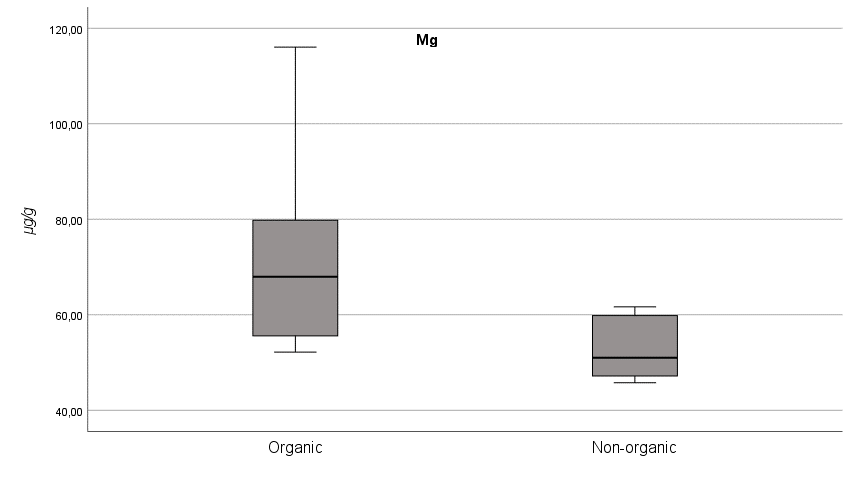


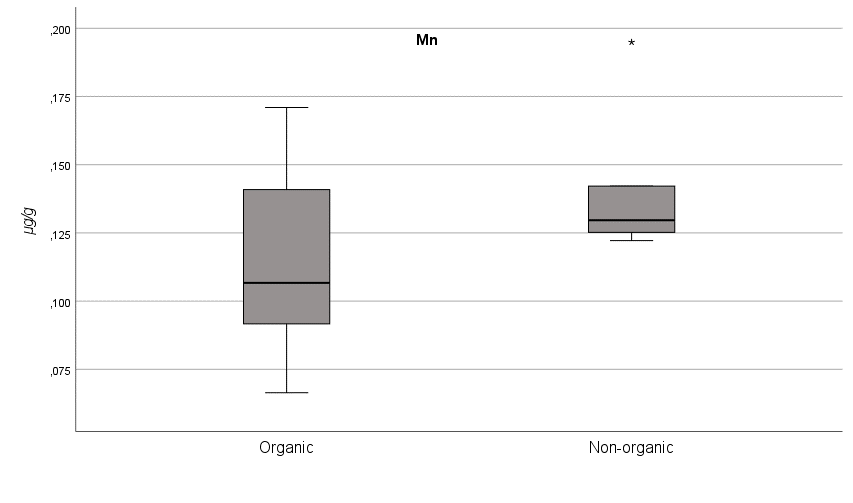


e)

d)


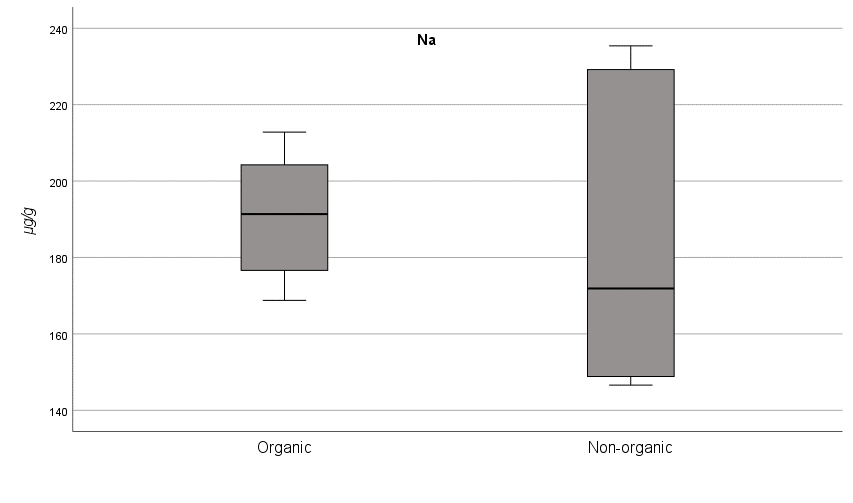


f)


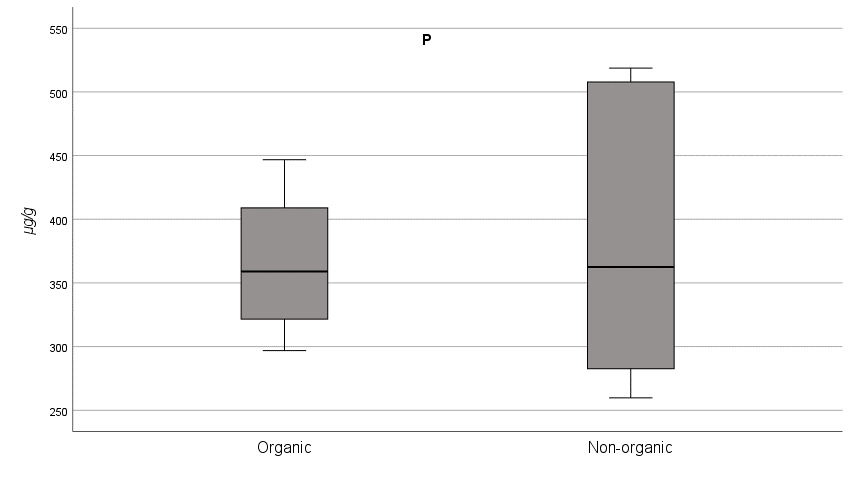


**Figure S6.** Box plots Ca (a), K (b), Mg (c), Mn (d), Na (e), P (f) concentrations in formula milks according to the production system (organic or non-organic).
